# Treatment of Class II Division 2 Malocclusion Using the Forsus Fatigue Resistance Device and 5-Year Follow-Up

**DOI:** 10.1155/2016/3168312

**Published:** 2016-02-29

**Authors:** Ezgi Atik, Ilken Kocadereli

**Affiliations:** Department of Orthodontics, Faculty of Dentistry, Hacettepe University, 06100 Ankara, Turkey

## Abstract

This case report presents the treatment of a 14-year-and-8-month-old boy with Class II division 2 mandibular retrusion, severe deep bite, and concave profile. The Forsus fatigue resistance device (FRD) was effective in correcting both skeletal and dental parameters. At 5-year posttreatment follow-up, the teeth were well aligned and the occlusion was stable. FRD application with appropriate treatment time can result with prominent changes in the facial profile and dentition, and the outcomes can be maintained at the long-term follow-up periods.

## 1. Introduction

Class II malocclusion is one of the most frequent problems encountered in orthodontics [1]. This malocclusion is described as a distal relationship of the mandible related to the maxilla with a combination of different dental and skeletal components which can affect facial aesthetics and functional status adversely [2].

The most common characteristic of Class II malocclusion is mandibular retrognathia rather than maxillary protrusion according to McNamara [3]. Functional orthopedic appliances are mostly used to treat Class II malocclusion originated from mandibular retrusion [4, 5]. Appliance selection can involve removable or fixed functional appliances according to the existing anteroposterior discrepancy, cooperation, and growth period of the patient. Nongrowing patients with Class II mandibular retrusion are mostly treated with fixed functional appliances which do not require the patient's collaboration [6–8].

One of the most preferred compliance free fixed functional appliances is Forsus Fatigue Resistant Device (FRD, 3M Unitek, Monrovia, Calif) which is used for the correction of Class II malocclusion with permanent dentition stage [9]. FRD can apply consistent forces with nickel-titanium coil springs, and the force level can be modified by the clinician [10]. In the literature, favorable dentoalveoler effects have been presented during postpubertal growth period by interarch fixed functional appliances [9–11]. The main functions of the FRD appliance are restraining sagittal maxillary growth, stimulating mandibular growth, inducing mesial movement of the mandibular arch, and distal movement of the maxillary arch [6].

The aim of this case report is to present both the effects of the FRD and 5 years' posttreatment stability on Class II division 2 patient with mandibular retrusion, severe deep bite, and concave profile.

## 2. Case Presentation

**Figure 1:**
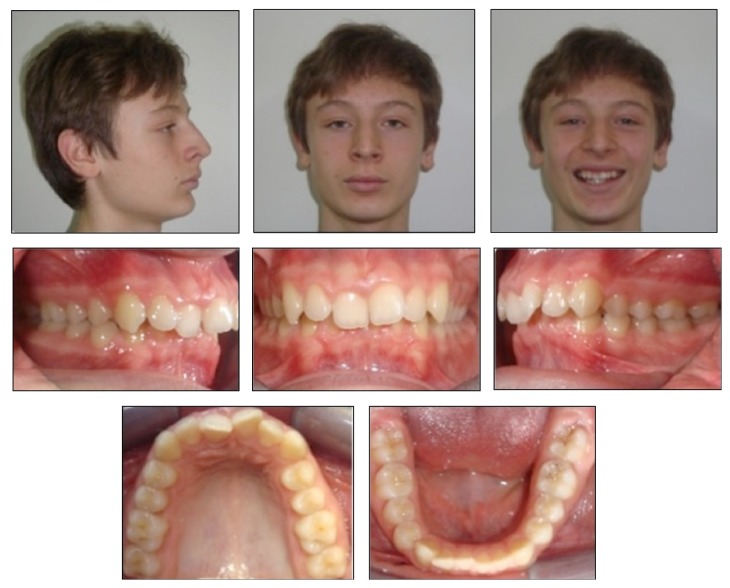
Pretreatment facial and intraoral photographs.

A 14-year-and-8-month-old Caucasion boy referred to our clinic with a chief complaint of irregular anterior teeth and backward positioned mandibular arch. Extraoral clinical examination indicated concave profile with prominent chin, decreased anterior facial height, and deep labiomental sulcus. The intraoral examination showed cusp to cusp canine and Class II molar relationships in both right and left segments with severe overjet (7.8 mm) and deep bite (7.5 mm). The maxillary and mandibular arch-length deficiencies were 3.8 and 2.5 mm, respectively (Figure 1).

**Figure 2:**
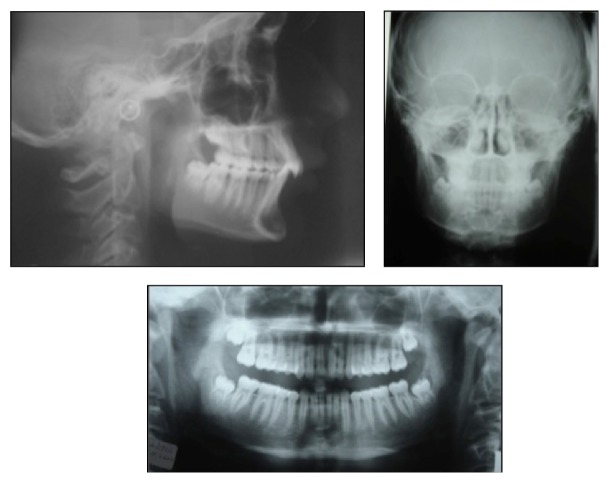
Pretreatment lateral, frontal, and panoramic radiographs.

Examination of the lateral cephalometric radiograph indicated normal positioned maxilla (SNA: 81°), retrognathic mandible (SNB: 75.6°), skeletal Class II malocclusion (ANB: 5.4°), and horizontal growth pattern (GoGnSN: 22.9°). The upper incisors were retroclined (U1-FH: 104.8°) and the lower incisors were proclined (IMPA: 101.1°). Panoramic radiographic evaluation showed permanent dentition with all teeth present including the third molars in all quadrants. Anteroposterior radiograph revealed no skeletal asymmetry (Figure 2).

### 2.1. Treatment Objectives

The treatment objectives were the following: (1) expansion of the dentally constricted maxillary arch; (2) protrusion of the severely retroclined upper incisors; (3) resolving the crowding of the maxillary and mandibular arch; (4) correction of severe deep bite; (5) establishing Class I canine and molar relationships; (6) obtaining normal overjet and overbite; and (7) improvement of the patient's facial esthetic.

### 2.2. Treatment Alternatives

The first treatment alternative was the orthognathic surgery with mandibular advancement and genioplasty. However the patient was unwilling to undergo the surgery. Therefore nonextraction orthodontic treatment protocol including interarch Class II mechanics was chosen for the treatment of the patient.

### 2.3. Treatment Progress

**Figure 3:**
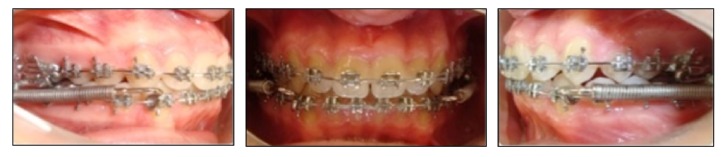
Progress intraoral photographs after application of the Forsus FRD.

At the beginning of the orthodontic treatment, maxillary dentoalveolar constriction was treated with Quad-helix appliance. When the desired maxillary expansion was achieved, upper incisors were bonded, leveled, and protruded with leveling and protrusion utility arches. Afterwards, upper and lower arches were bonded with 0.018-inch Roth prescribed appliances (Forestadent, Pforzheim, Germany). Leveling and aligning stage started with 0.014-inch Ni-Ti and continued with 0.016-inch Ni-Ti, 0.016 × 0.016-inch Ni-Ti, 0.016 × 0.016-inch stainless steel (SS), 0.016 × 0.022-inch SS, and 0.017 × 0.025-inch SS archwires, respectively. In the upper arch, a transpalatal arch was applied to minimize the potential transverse side effects of the FRD appliance. After leveling and alignment were achieved, the FRD appliances with a 32 mm length of rod were inserted bilaterally when 0.017 × 0.025-inch SS archwires were inserted at both maxillary and mandibular arches (Figure 3). A lingual arch was placed and the SS archwire was cinched back in the lower arch at the stage of FRD insertion. In addition to these, extra buccal root torque was applied to the lower incisors to limit the buccal inclination of the lower incisors. The lower parts of the FRD appliance were placed on the distal to the mandibular canine teeth.

**Figure 4:**
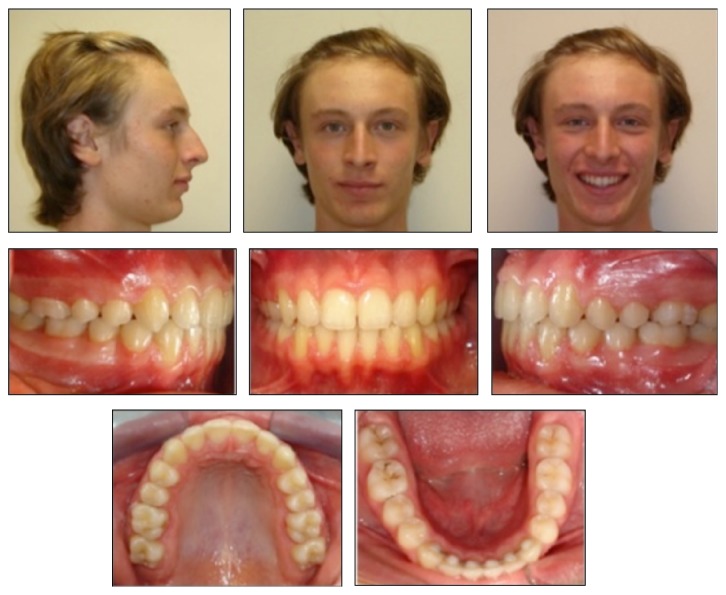
Posttreatment facial and intraoral photographs.

Activation of the FRD appliance was applied with 4-week intervals until a super Class I canine and molar relationships were obtained and overjet was eliminated. Active FRD application took 4 months. Total active orthodontic treatment period was 16 months. After obtaining ideal overjet, overbite and a functional interdigitation, brackets were removed and the retention period began (Figure 4). Increase in the mentolabial angle was significant in posttreatment facial photograph (Figure 4).

During the retention period, the patient was instructed to wear Hawley retainer which had anterior bite plane in the maxillary arch and in the lower Hawley retainer with fixed mandibular lingual retainer for 12 months all day.

### 2.4. Treatment Results

**Figure 5:**
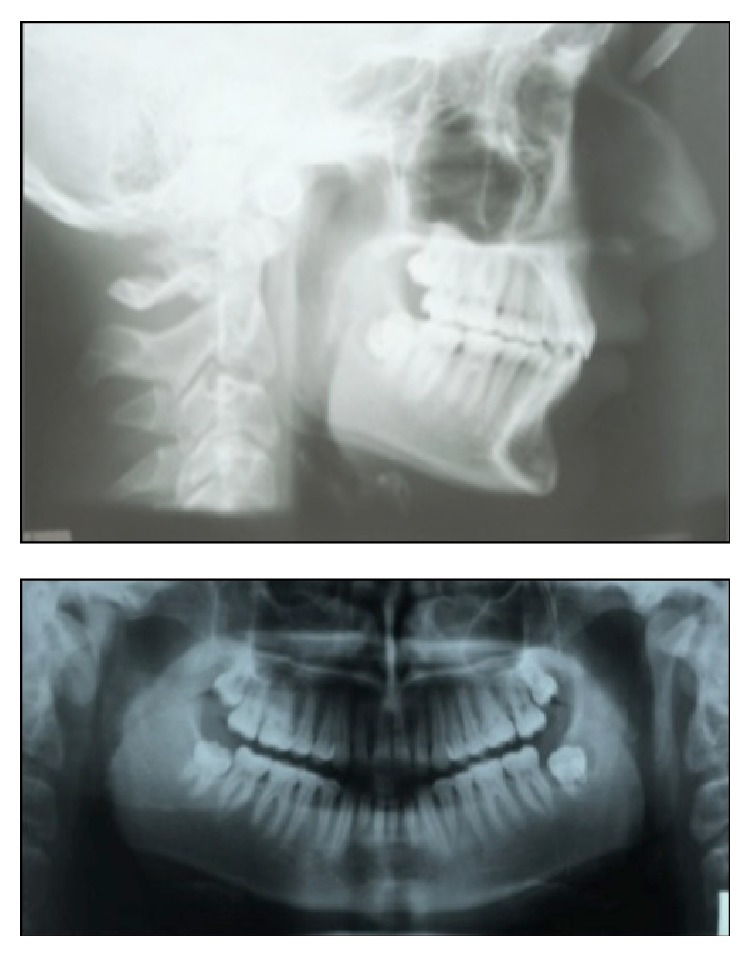
Posttreatment lateral and panoramic radiographs.

**Figure 6:**
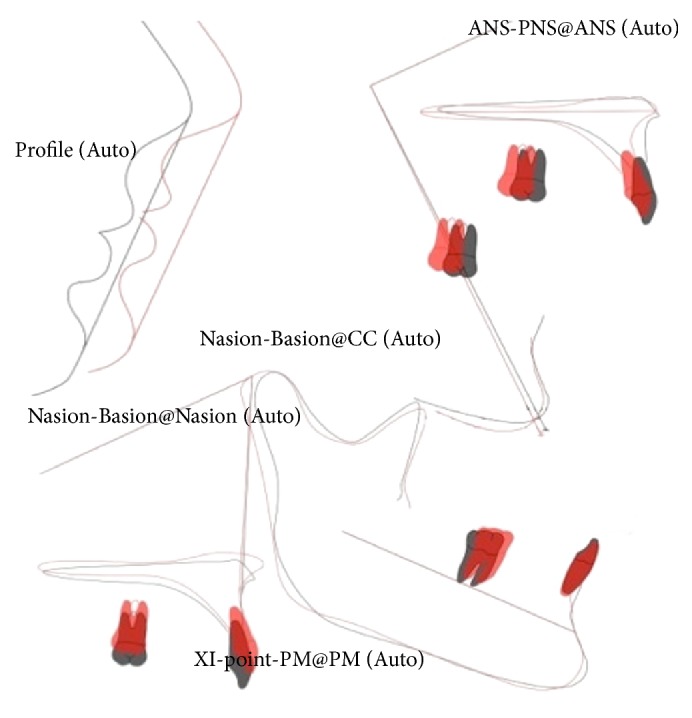
Superimposition (black line, pretreatment; red line, posttreatment).

Cephalometric measurements at the pretreatment, posttreatment and postretention (5 years' follow-up) periods are given in Table 1. The results indicated improvement in both skeletal and dental parameters. At the end of treatment, overjet was reduced from 7.8 mm to 2.6 mm and overbite reduced from 7.5 mm to 2.2 mm. Cephalometric superimposition indicated downward and forward movement of the mandibular dentoalveolar arch and backward movement of the maxillary dentoalveolar arch. ANB angle decreased from 5.4 degrees to 2.5 degrees. Convexity decreased and the prominency of labiomental fold diminished. The posttreatment panoramic radiograph showed no alveolar bone loss or apical root resorption (Figures 5 and 6).

**Figure 7:**
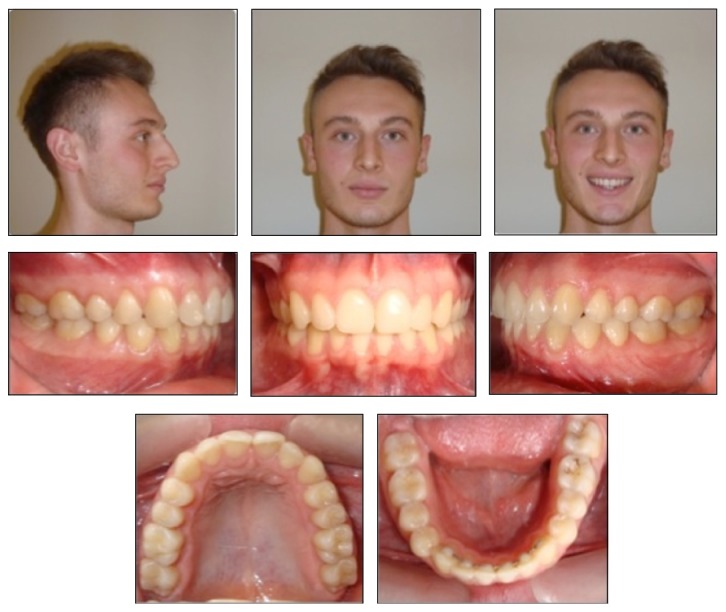
Facial and intraoral photographs after 5 years of retention.

Posttreatment follow-ups were carried out after 5 years (Figure 7). Intraoral photographs showed that teeth were well aligned and the occlusion was stable. Cephalometric measurements indicated that maxillary incisors were slightly labially inclined, however mandibular incisors were unchanged (Table 1). Third molars were in good positions as seen in the posttreatment panoramic radiograph; however the patient extracted both maxillary and mandibular third molars.

## 3. Discussion

In adult patients with Class II mandibular retrusion, either camouflage or orthognathic surgical treatment is carried out depending upon the severity of maxillomandibular discrepancy [12]. A surgical procedure was suggested to improve the facial esthetic; however the patient declined this approach. Extraction treatment was not suitable since the patient had both severe deep-bite and concave profile which could become worsen the occlusion and the profile. Therefore it was decided to apply nonextraction orthodontic treatment including Class II inter-arch mechanics.

FRD appliance is usually recommended for the Class II malocclusions especially in patients with mandibular dentoalveolar retrusion. This appliance can lead mandibular growth and favorable dentoalveolar changes in patients at or before the peak phase of pubertal growth [13, 14]. On the other hand, mostly dental changes are encountered for the patients at postpubertal period [15]. However, in this case report, the increase of the SNB angle was nearly 3 degrees and the patient showed slight forward mandibular displacement after the treatment. This result can be correlated with the minimal residual growth of the patient during orthodontic treatment. The dentoalveolar changes were evident at both maxillary and mandibular arches (Table 1). Maxillary incisors and first molars demonstrated distal movement and intrusion. Mandibular first molars showed mesial movement and extrusion, and lower incisors exhibited proclination. The correction of the overjet was achieved by both retroclination of the upper incisors and protrusion of the lower incisors. Similar dental changes are also reported by the other studies [6, 10].

Application of negative torque to the lower incisors and a lingual arch did not eliminate the unfavorable lower incisor protrusion. Even with these anchorage mechanics, mandibular incisors were proclined by 5 degrees. Increase in the mandibular incisor inclination is a similar common finding of fixed functional appliances as shown by the other studies [9, 16]. To eliminate this side effect of the FRD appliance, it could be effective to use miniscrew anchorage as shown by Aslan et al. [17]. Furthermore, mandibular rectangular archwires of greater size and addition of negative torque in the lower incisor region can be considered.

The patient was followed up for 5 years. Stable treatment results were obtained with the FRD in this young adult patient, and this result was in accordance with the outcomes of the studies observing stability after fixed functional appliance systems. Bock et al. [12] reported stable molar and canine relationships in Class II adult patients treated with Herbst appliance. Ruf and Pancherz [18] found fewer relapse in postpeak patients treated with Herbst/multibracket appliance system. Gao et al. [19] evaluated the effects and the stability of FRD appliance treatment and concluded relatively stable results 2 years after treatment. In a case report, Zhang et al. [20] reported a stable and functional occlusion using Forsus FRD in a young patient with two congenitally missing mandibular incisors after 42 months of retention. The stable results in the long-term follow-up periods in our case report may be related with different factors including finishing the treatment with stable interdigitation in permanent dentition and the patient's postpeak growth.

## 4. Conclusion

Fixed functional appliance (FRD) application with appropriate treatment time resulted in prominent changes in the facial profile and dentition. The stability of the outcomes was maintained at the long-term follow-up periods.

## Figures and Tables

**Table 1:** Mean values of the measurements at pretreatment (T0), posttreatment (T1), and 5 years' after retention (T2).

Measurement	Norm	Pretreatment (T0)	Posttreatment (T1)	5 years' after retention (T2)
SNA°	82°	81°	81°	79.6°
SNB°	80°	75.6°	78.5°	77.2°
ANB°	2°	5.4°	2.5°	2.3°
Convexity (mm)	0.8 mm	3.2 mm	0.2 mm	−0.5 mm
GoGnSN°	32°	22.9°	21.6°	21.8°
U1-FH°	111°	104.8°	103.4°	111.3°
U1-NA°	22°	14.3°	17.9°	23.5°
IMPA°	90°	101.1°	106°	107°
L1-NB°	25°	19.5°	26.1°	26.7°
Interincisor angle°	130°	140.8°	130.7°	127.9°
U6-PTV (mm)	17.7 mm	22.1 mm	16.7 mm	19.4 mm
Overjet (mm)	2.5 mm	7.8 mm	2.6 mm	2.6 mm
Overbite (mm)	2.5 mm	7.5 mm	2.2 mm	2.6 mm
Low.Lip-E (mm)	−2.0 mm	−3.9 mm	−6 mm	−5.9 mm
